# SIN3A Regulates Porcine Early Embryonic Development by Modulating *CCNB1* Expression

**DOI:** 10.3389/fcell.2021.604232

**Published:** 2021-02-22

**Authors:** Lei Luo, Yanna Dang, Yan Shi, Panpan Zhao, Yunhai Zhang, Kun Zhang

**Affiliations:** ^1^Laboratory of Mammalian Molecular Embryology, Assisted Reproduction Unit, Department of Obstetrics and Gynecology, Sir Run Run Shaw Hospital, School of Medicine, College of Animal Sciences, Zhejiang University, Hangzhou, China; ^2^Anhui Provincial Laboratory of Local Livestock and Poultry Genetical Resource Conservation and Breeding, College of Animal Science and Technology, Anhui Agricultural University, Hefei, China

**Keywords:** pig, cattle, embryo, preimplantation, SIN3A, CCNB1

## Abstract

SIN3A is the central scaffold protein of the SIN3/histone deacetylase (HDAC) transcriptional repressor complex. SIN3A participates in the mouse preimplantation development by fine-tuning *HDAC1* expression. However, it remains unresolved if this functional significance of SIN3A was conserved in other mammals. Herein, RNA-seq results show a large amount of *SIN3A* mRNA is present in oocytes and early embryos prior to embryonic genome activation and a low amount thereafter, suggesting a maternal origin of SIN3A in pigs, cattle, mice, and humans. Interestingly, immunofluorescence data show that SIN3A protein level peaks at four-cell stage in pigs compared with morula stage in cattle. SIN3A depletion in early embryos causes a developmental arrest at two-cell stage in pigs but does not affect bovine early embryonic development. In contrast with mouse data, SIN3A depletion results in only a slight decrease and even no difference in HDAC1 expression in porcine and bovine early embryos, respectively. In addition, HDAC1 knockdown does not cause two-cell block but leads to a reduced blastocyst rate. By using unbiased RNA-seq approach, we found that *Cyclin B1* (*CCNB1*) transcript level is dramatically reduced. Moreover, CCNB1 knockdown results in a similar phenotype as SIN3A depletion. Injection of exogenous *CCNB1* mRNA into SIN3A-depleted embryos could partly rescue embryonic development to pass two-cell stage. In conclusion, our results indicate SIN3A plays an essential role in porcine early embryonic development, which probably involves the regulation of *CCNB1* expression.

## Introduction

Life is initiated with the fusion of sperm and oocyte. Immediately following fertilization, early embryos undergo substantial epigenetic reprogramming to ensure the conversion of cell potency from extreme differentiation to totipotent status. During this process, the transcriptional apparatus is generally inactive until embryonic genome activation (EGA) takes place (mouse: two-cell; pig, and human: four to eight cells; cattle: 8–16 cells) (Schultz et al., 2018; Schulz and Harrison, 2019). Meanwhile, the control of early embryonic development is gradually switched from the oocyte to the embryo itself, whose transition is also termed maternal-embryonic transition (MET). A unique characteristic of early embryonic development is its reliance on maternal-stored factors, especially prior to EGA (Eckersley-Maslin et al., 2018). Maternal factors, including mRNAs and proteins, are critical to key biological events occurring in preimplantation development, including chromatin reprogramming (Inoue et al., 2017; Kannampuzha-Francis et al., 2017), EGA (De Iaco et al., 2017; Hendrickson et al., 2017), and maintenance of genome stability (Xu et al., 2017). However, the mechanisms underlying these regulations remain poorly understood, especially in domestic animals.

SIN3/histone deacetylase (HDAC) chromatin complex has long been recognized as a transcriptional repressor. As the master scaffold protein of the complex, SIN3A does not possess DNA binding ability by itself, however, could regulate chromatin structure and affect gene expression by recruiting other epigenetic components, including HDAC1, HDAC2, and Ten–eleven translocation 1 (Tet1) (Tiana et al., 2018; Gambi et al., 2019; Monteleone and Poli, 2019). Moreover, growing evidence indicates SIN3/HDAC complex is not only involved in transcriptional repression but activation of gene expression (Icardi et al., 2012; Laugesen and Helin, 2014). HDAC1 and HDAC2 are two highly homologous lysine deacetylases, which exist together in multiple other chromatin complexes, namely nucleosome remodeling and deacetylase (NuRD) (Xue et al., 1998) and corepressor for the REST/NRSF transcription factor (CoREST) (You et al., 2001). These complexes are conserved from yeast to human (Sheikh and Akhtar, 2019).

SIN3A is expressed abundantly in mouse oocytes and early embryos (Jimenez et al., 2015; Zhao et al., 2019). Single-cell RNA-seq analyses further revealed that it is a hub gene of the transcriptome networks in both mouse and human preimplantation embryos, suggesting a key functional role (Xue et al., 2013). Indeed, none of *mSin3a^–/–^* blastocyst is obtained at approximately embryonic day 6.5 from *mSin3a*^±^ mouse intercrosses (McDonel et al., 2012). Inhibition of *Sin3a* expression *via* RNAi during oocyte maturation leads to developmental arrest at the two-cell stage and global gene expression profile is disturbed (Jimenez et al., 2015). We also found *Sin3a* deficiency in mouse early embryos causes embryonic block at morula stage, which is mediated through regulation of *Hdac1* (Zhao et al., 2019). Nonetheless, the functional requirement for SIN3A has been primarily conducted in murine model and has not been addressed in other species, including domestic animals.

Herein, we compared the expression pattern of SIN3A in four representative species. Results show that SIN3A transcript level is dynamic and exhibits a pattern of maternal-effect gene during preimplantation development in pigs, cattle, mice, and humans, whereas SIN3A protein displays a stage-specific and species-specific pattern. Knocking down SIN3A causes embryonic arrest at two-cell stage, resulting in a slight decrease in HDAC1 level and an increase in H3K5ac level in porcine early embryos. However, SIN3A depletion does not affect HDAC1 expression and developmental competence of bovine early embryos, indicating species-specific role of SIN3A during early embryonic development. We further found that the phenotype of porcine SIN3A-depleted embryos cannot be attributed to the reduced HDAC1 since HDAC1 knockdown (KD) embryos could pass through the two-cell stage. Moreover, RNA-seq results indicate a significant reduction in *Cyclin B1* (*CCNB1*) mRNA level. Functional evidence proves that the phenotype of CCNB1 KD embryo mimics the one of SIN3A-depleted embryos and the exogenous *CCNB1* mRNA could partly rescue the SIN3A-deficient embryos to pass through the two-cell stage. In sum, we propose that SIN3A is required for porcine early embryonic development, likely through the regulation of *CCNB1* expression.

## Materials and Methods

All chemical reagents were purchased from Sigma-Aldrich (St. Louis, MO, United States) unless stated elsewhere.

### *In vitro* Maturation of Porcine Oocyte

Porcine *in vitro* maturation (IVM) was performed based on procedures as described previously (Ding et al., 2017; Cao et al., 2019, 2020). Peripuberty porcine ovaries of crossbreeds (Landrace × Yorkshire × Duroc) were collected from a local slaughterhouse and transported to the laboratory at 28–35°C in physiological saline solution. Ovaries were quickly washed in saline, and the follicles with 3 to 6 mm in diameter were aspirated using a sterile 10-ml syringe. Cumulus-oocyte complexes (COCs) within the follicular fluid were settled down at 38.5°C for 15 min. COCs with more than three layers of cumulus cells and homogeneous ooplasm were selected for subsequent experiments using a stereomicroscope. After washing three times in IVM medium, appropriately 50 COCs were transferred to 400 μl IVM medium (TCM-199 supplemented with 5% FBS, 10% porcine follicular fluid, 10 IU/ml eCG, 5 IU/ml hCG, 100 ng/ml L-cysteine, 10 ng/ml EGF, 0.23 ng/ml melatonin, 2.03 × 10^–5^ ng/ml LIF, 2 × 10^–5^ ng/ml IGF, 1.4 × 10^–5^ ng/ml FGF2, 100 U/ml penicillin, and 100 mg/ml streptomycin) covered with mineral oil in four-well plates and cultured for 42–44 h at 38.5°C, 5% CO_2_ in air with saturated humidity. After maturation, cumulus cells surrounding oocytes were removed by gentle pipetting in 1 mg/ml hyaluronidase in DPBS without Ca^2+^ and Mg^2+^. Only the matured oocytes that possess an extruded first polar body and uniform ooplasm were selected for subsequent experiments.

### Parthenogenetic Activation of Porcine Oocytes

As described previously (Ding et al., 2017), oocytes were washed with activation medium containing 280 mM mannitol, 0.1 mM CaCl_2_, 0.1 mM MgCl_2_, and 0.01% polyvinyl alcohol. Then oocytes were treated with two pulses of direct current (1.56 kV/cm for 80 μs) by using cell fusion instrument (CF-150B, BLS, Hungary) in a chamber covered with activation medium. Subsequently, embryos were washed three times in porcine zygote medium (PZM-3), followed by 4 h of incubation in the chemically assisted activation medium (PZM-3 supplemented with 10 μg/ml cycloheximide and 10 μg/ml cytochalasin B). Embryos were then washed three times with PZM-3 medium and cultured in fresh PZM-3 medium at 38.5°C, 5% CO_2_ in air with saturated humidity.

### Bovine *in vitro* Embryo Production

Bovine IVM, IVF, and IVC were conducted based on protocols published previously (Zhang et al., 2016, 2018; Wang et al., 2020). In brief, COCs with more than three layers of cumulus cells were collected from abattoir-origin ovaries. The maturation medium consists of Medium-199 (Sigma-Aldrich, M4530) supplemented with 10% FBS (Gibco-BRL, Grand Island, NE, United States), 1 IU/ml FSH (Sansheng Biological Technology, Ningbo, China), 1 mM Na Pyruvate (Thermo Fisher Scientific, Waltham, MA, United States), 2.5 mM GlutaMAX (Thermo Fisher Scientific, Waltham, MA, United States), and 10 μg/ml gentamicin. COCs were cultured at 38.5°C and 5% CO_2_ in humidified air for 22–24 h. Upon maturation, COCs were cocultured with spermatozoa purified from frozen-thawed semen by 90% Percoll gradient with 100 COCs placed per well using four-well plates. IVF condition was 38.5°C and 5% CO_2_ for 9–12 h. Then, enclosing cumulus cells were removed from putative zygotes by pipetting up and down with 1 mg/ml hyaluronidase. Embryos were incubated with BO-IVC medium (IVF Bioscience, Falmouth, United Kingdom) at 38.5°C and 5% CO_2_ in humidified air.

### Real-Time Quantitative Polymerase Chain Reaction

Total RNA was extracted from 20 oocytes or 15 embryos using the RNeasy Mini Kit (Qiagen, Hilden, Germany) and reverse transcribed using QuantiTect Reverse Transcription Kit (Qiagen, Hilden, Germany). Quantitative polymerase chain reaction (qPCR) was conducted using FastStart SYBR Green Master mix (Roche, Rotkreuz, Switzerland) according to manufacturer’s protocol and was run on a StepOne Plus Real-Time PCR System (Applied Biosystems, Foster City, CA, United States). Gene expression was analyzed by employing relative quantification and 2^–Δ^ ^Δ^ ^*CT*^ method, and quantification was normalized to *H2AFZ* (Ding et al., 2017). Primers used in the present study are listed in Table 1.

**TABLE 1:** PCR primers used in the current study.

Name	Sequences (5′–3′)
*H2AFZ*	FP: CCAAGACAAAGGCGGTTTCC RP: TGGCTGGTCGTCCTAGATTTC
*HDAC1*	FP: TGCTAAAGTATCACCAGAGGGT RP: AGCCCCAATATCCCGTAGGT
*HDAC2*	FP: AGCTTTCAACTGGTGGCTCA RP: ACTCAAGGATGGCAAGCACA
*CCNB1*	FP: AGCTAGTGGTGGCTTCAAGG RP: GCGCCATGACTTCCTCTGTA

### Microinjection

Microinjection of small interfering RNA (siRNA)/mRNA into porcine or bovine oocytes/embryos were performed as published previously (Zhang et al., 2016, 2018; Ding et al., 2017; Wang et al., 2020). To deplete endogenous *SIN3A* mRNA, siRNAs of *SIN3A* were microinjected into the cytoplasm of MII stage oocytes in pigs prior to parthenogenetic activation or to putative zygotes in cattle. Three siRNAs were purchased to target different sites of porcine or bovine *SIN3A*, and non-specific siRNA was used as a negative control (GenePharma, Shanghai, China; Table 2). For porcine oocytes, microinjection was performed in oocyte manipulation medium (TCM199 with 2% FBS and 7.5 μg/ml cytochalasin B) on the heated stage of an inverted microscope (Olympus, Japan). For bovine zygotes, microinjection was performed in manipulation of medium under room temperature (RT). Approximately 10 pl siRNA solution was microinjected into the cytoplasm of oocytes or zygotes. The manipulated porcine oocytes were parthenogenetically activated after recovering for 30 min in PZM-3 and then cultured *in vitro*.

**TABLE 2:** siRNA information.

Name	Sequences (5′–3′)
Pig SIN3A siRNA 1	Sense: CCAAGUGAAGCUACAGUUUTTAntisense: AAACUGUAGCUUCACUUGGTT
Pig SIN3A siRNA 2	Sense: GGAUUCUUCUAUGGCAGAUTTAntisense: AUCUGCCAUAGAAGAAUCCTT
Pig SIN3A siRNA 3	Sense: CCAAGGUCCUGAGAUCUAATTAntisense: UUAGAUCUCAGGACCUUGGTT
Pig HDAC1 siRNA 1	Sense: GCUCCAUCCGCCCAGAUAATTAntisense: UUAUCUGGGCGGAUGGAGCTT
Pig HDAC1 siRNA 2	Sense: GGAGAGUACUUCCCAGGAATTAntisense: UUCCUGGGAAGUACUCUCCTT
Pig HDAC1 siRNA 3	Sense: GGAGAUCCCUAAUGAGCUUTTAntisense: AAGCUCAUUAGGGAUCUCCTT
Pig HDAC2 siRNA 1	Sense: GCAUAACUUGCUGCUAAAUTTAntisense: AUUUAGCAGCAAGUUAUGCTT
Pig HDAC2 siRNA 2	Sense: GCAAAUACUAUGCUGUCAATTAntisense: UUGACAGCAUAGUAUUUGCTT
Pig HDAC2 siRNA 3	Sense: GGAGCAAAGAAAGCUAGAATTAntisense: UUCUAGCUUUCUUUGCUCCTT
Cattle SIN3A siRNA 1	Sense: GCAGAGCUCUAGCAGUCAUTTAntisense: AUGACUGCUAGAGCUCUGCTT
Cattle SIN3A siRNA 2	Sense: CCACCUUUGUUAGUUCCAATTAntisense: UUGGAACUAACAAAGGUGGTT
Cattle SIN3A siRNA 3	Sense: CCCAUGAGCGUGUAAGCAATTAntisense: UUGCUUACACGCUCAUGGGTT

### Immunofluorescence Staining

Oocytes or embryos were washed in DPBS, fixed in 4% paraformaldehyde (PFA) solution for 15 min, permeabilized with 1% Triton X-100 in DPBS for 30 min at RT, and blocked with 2% BSA in DPBS at RT for 1 h. The samples were incubated in the blocking solution containing primary antibodies overnight at 4°C. Following four washes, the samples were incubated for 1 h in the blocking solution containing secondary antibodies in the dark at 37°C. Antibodies used in the current study were mouse anti-SIN3A (1:200, Santa Cruz, sc-5299), mouse anti-HDAC1 (1:200, Cell Signaling Technology, 5356), rabbit anti-HDAC2 (1:200, Abcam, ab32117), and H4K5ac (1:200, Millipore, 07-327). After three washes, the samples were counterstained for 10 min in 4,6-diamidino-2-phenylindole dihydrochloride (DAPI) solution and loaded onto glass slides and covered with a glass coverslip. Samples were imaged using inverted fluorescence microscope (Olympus, Japan). Groups of embryos stained without primary antibodies or secondary antibodies or both antibodies were used as negative controls to validate the specificity of the reaction. At least 10 oocytes/embryos were processed for each group.

The signal intensity for immunofluorescence was quantified using ImageJ software (ImageJ 1.43u, NIH) as described previously (Ding et al., 2017). Briefly, the nuclear area was encircled based on DAPI signal. The signal intensity of each protein was measured, and the cytoplasmic area was measured for normalization. The average signal intensity of the nuclear areas was calculated by subtracting the average intensity of the background areas.

### RNA-Seq

Two-cell stage embryos (32 h after parthenogenetic activation) were harvested from NC and KD groups (*n* = 3; 20 embryos/group/replicate). Total RNA isolation was performed using PicoPure RNA Isolation Kit (Thermo Fisher Scientific, Waltham, MA, United States) based on the manufacturer’s manual. Sequencing libraries were constructed with NEBNext Ultra RNA Library Prep Kit for Illumina (New England Biolabs, Ipswich, MA, United States) according to the manufacturer’s instructions. Libraries were sequenced using Illumina HiSeq X Ten (Illumina, San Diego, CA, United States) by Novogene Co. Ltd. The sequencing reads were assigned directly to porcine transcripts (Sscrofa11.1) and quantified using Salmon^1^ (Patro et al., 2017; Sahraeian et al., 2017). Analysis of differential gene expression was carried out using the DESeq2 package [adjusted *P* < 0.05 and Log_2_ (fold change) > 0.8 or ≤0.8] (Love et al., 2014). For the profile of SIN3A in different species, RNA-seq datasets were downloaded from GEO database and mapped to genome using Hisat2^2^. The gene expression level was then calculated with Cufflinks^3^.

### *In vitro* Transcription

Total RNA was extracted from mouse ovary and reverse transcribed to obtain a cDNA library. *CCNB1* mRNA primers containing the T7 promoter sequence were designed. The *CCNB1* sequence was amplified from the cDNA library using high fidelity DNA polymerases and recovered by gel electrophoresis to generate the wild-type template. After adding the poly A-tail to the wild-type cDNA template, the amplicon was ligated with the pMD18-T vector and transformed into *Escherichia coli* DH5α-competent cells, and single colonies were selected for identification and screening by PCR and sequencing after being cultured. The plasmid was extracted, linearized, and subject to *in vitro* transcription. The resultant RNA was purified according to the instructions of the *in vitro* transcription kit (mMESSAGE mMACHINE T7 ULTRA Transcription Kit). The mRNA concentration at the time of microinjection was 700 ng/μl.

### Statistical Analysis

All experiments were carried out at least three times. Before statistical analysis, the percentage data underwent arc-sin transformation; nonetheless, results were still presented as untransformed. Independent-sample *t* test was used to analyze differences between two groups and one-way analysis of variance (ANOVA) to analyze multiple comparison tests. The data were presented as means ± SEM. *P* < 0.05 was considered to be statistically significant.

## Results

### SIN3A mRNA Abundance Decreased Sharply During Embryonic Genome Activation in Mammals

**FIGURE 1:**
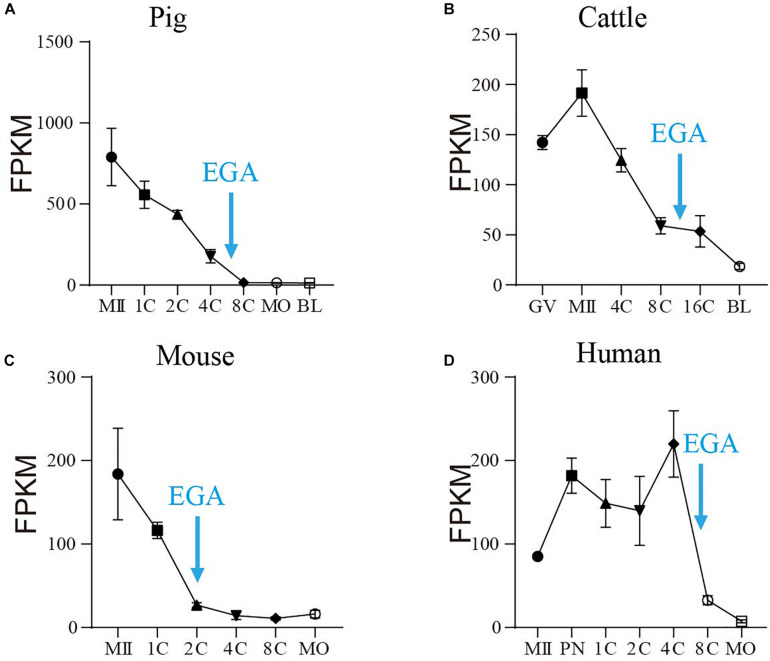
The mRNA profile of SIN3A during oocyte maturation and early embryonic development in pigs, cattle, mice, and humans. **(A)** Pig. **(B)** Cattle. **(C)** Mice. **(D)** Human. All the SIN3A mRNA expression data in different species were collected from published databases. GV, germinal vesicle; MII, metaphase II; PN, pronucleus; 1C, one-cell; 2C, two-cell; 4C, four-cell; 8C, eight-cell; 16C: 16-cell; MO, morula; BL, blastocysts. The blue arrow indicates the time of embryonic genome activation (EGA) for each species.

RNA-seq analyses revealed that SIN3A is a stage-specific hub gene expressed in both human and mouse early embryos (Xue et al., 2013), suggesting a critical role of SIN3A in preimplantation development. To test if the expression pattern of SIN3A preserved in mammals, we first compared *SIN3A* mRNA level during preimplantation development in four mammalian species (pigs, cattle, mouse, and human) using public RNA-seq datasets (Xue et al., 2013; Graf et al., 2014; Kong et al., 2020). Results showed *SIN3A* mRNA was detectable throughout oocyte maturation and preimplantation development in a dynamic manner in all species examined (Figures 1A–D). In general, *SIN3A* mRNA was present abundantly in oocytes with a dramatic decrease in abundance after fertilization in pigs, cattle, and mice. However, *SIN3A* mRNA abundance increased after fertilization, maintained through four-cell stage and thereafter decreased dramatically in humans. Interestingly, for all species examined, the mRNA abundance was significantly reduced during the stage corresponding to EGA in each specie (four- to eight-cell stage for pigs and humans, eight- to 16-cell stage for cattle, and two-cell stage for mice) with lowest amount observed afterward, suggesting a maternal origin of *SIN3A*. These data pointed out that *SIN3A* mRNA profile exhibits a conserved pattern during mammalian preimplantation development and suggest a functional significance of SIN3A in early embryonic development prior to EGA.

### SIN3A Protein Profiles Exhibit Species-Specific Pattern During Early Embryonic Development in Mammals

**FIGURE 2:**
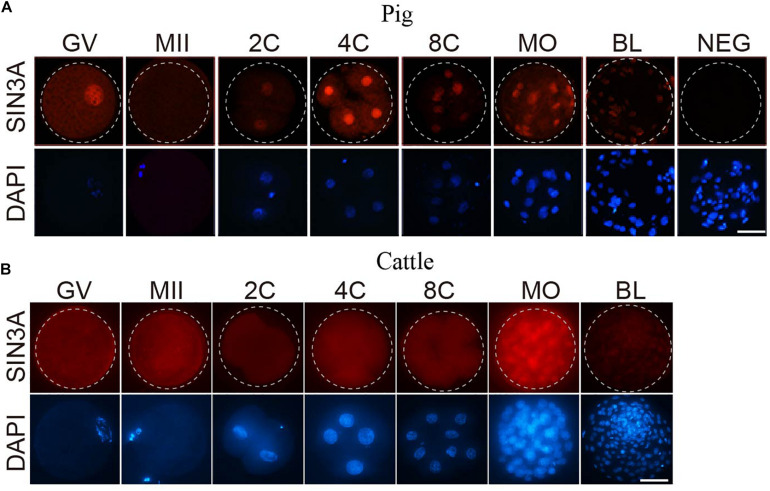
Immunofluorescence detection of SIN3A protein during oocyte maturation and embryonic development in pig and cattle. **(A)** Pig. **(B)** Cattle. SIN3A protein (red) was probed with mouse anti-SIN3A antibodies and detected by Alexa 594-conjugated goat anti-mouse antibodies. Nuclei (blue) were labeled with DAPI stain. The experiment was independently repeated three times with at least 10 oocytes or embryos per stage. Scale bar = 50 μM.

To test if there was corresponding change in SIN3A protein profiles, we performed immunofluorescence against SIN3A during preimplantation development in porcine and bovine embryos. We first validated the efficacy of the commercial antibody against SIN3A by analyzing SIN3A overexpressed mouse embryos. Results showed an obvious increase in SIN3A signal intensity in *SIN3A* mRNA-injected embryos relative to controls, indicating a robust specificity of the antibody used (Supplementary Figure 1). Immunofluorescent results revealed that a relatively small amount of SIN3A was observed in germinal vesicle (GV) stage oocytes and no detectable signal in MII oocytes, likely due to the remarkable dilution of nuclear content after GV breakdown (Figure 2A). Following fertilization, SIN3A protein can be easily detected in the nuclei from the beginning of two-cell stage, peaked at four-cell, and reduced sharply thereafter during preimplantation development in pigs (Figure 2A), suggestive of a critical role of SIN3A during two- to four-cell stage. However, we have found no restricted signal of SIN3A in bovine oocytes and preimplantation embryos until morula stage (Figure 2B), indicating species-specific pattern of SIN3A protein profiles.

### SIN3A Knockdown Blocked Porcine Early Embryonic Development at Two-Cell Stage

**FIGURE 3:**
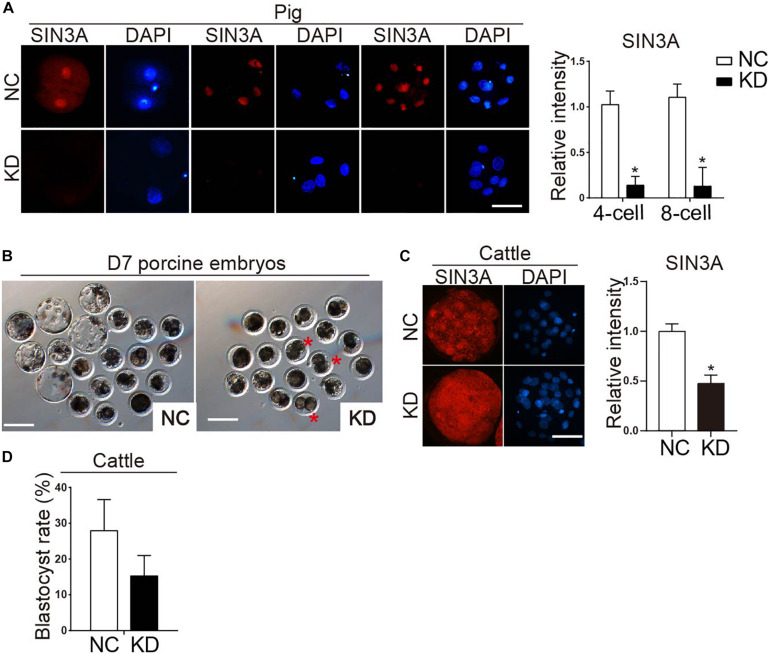
Effect of SIN3A knockdown on the developmental efficiency of porcine parthenogenetic and bovine *in vitro* fertilized embryos. **(A)** Immunofluorescence analysis of SIN3A knockdown efficiency in porcine two-, four-, and eight-cell embryos (left). NC represents the embryos injected with negative control siRNA, and KD represents the embryos injected with SIN3A-specific siRNAs. Three independent replicates were performed with 5–10 embryos/group/replicate analyzed. SIN3A (red), nuclei (blue). Scale bar = 50 μM. Average value of the signal intensities of SIN3A was assessed by densitometry (right). Data are expressed as mean ± SEM. *P < 0.05. **(B)** Representative pictures of NC and KD porcine embryos after *in vitro* culture for 7 days. Asterisk indicates abnormal embryos which appeared blocked at two- to four-cell stage in KD group. **(C)** Detection of knockdown efficacy of bovine SIN3A-specific siRNA at the morula stage. Three independent replicates were performed with six- to eight-cell embryos/group/replicate analyzed. Average value of the signal intensities of SIN3A was assessed by densitometry (right). **(D)** Blastocyst formation rate of bovine embryos after SIN3A KD. The rate of blastocysts at day 7 was recorded with no significant difference found between the NC and KD groups. Four independent replicates, all data were presented as mean ± SEM.

To determine the biological function of SIN3A in porcine early embryonic development, siRNAs against *SIN3A* were commercially obtained and microinjected into porcine-matured oocytes, followed by parthenogenetic activation. To validate the efficacy of *SIN3A* siRNAs, immunofluorescence staining was performed to examine SIN3A protein level in porcine preimplantation embryos (Figure 3A). Results confirmed that the SIN3A protein was nearly undetectable in KD groups relative to NC groups at the two-, four-, and eight-cell stages (Figure 3A, left), and the signal intensity of SIN3A decreased by more than 80% relative to control groups (Figure 3A, right), proving a robust efficacy of the siRNA used in porcine preimplantation embryos. Because no effective siRNA targeting untranslated regions (UTR) of *SIN3A* mRNA was available, we did not perform experiments to determine if exogenous SIN3A complementary RNA (cRNA) could rescue the development of SIN3A-depleted embryos.

To determine whether SIN3A KD affected porcine preimplantation embryo development, blastomere cleavage and blastocyst formation were monitored. Results showed that SIN3A KD did not affect the cleavage rate (Figure 3B and Table 3; NC vs. KD, 83.63 ± 3.63% vs. 80.08 ± 4.66%). However, the embryos deficient of SIN3A were severely affected in terms of blastocyst formation relative to control (Figure 3B and Table 3; NC vs. KD, 36.64 ± 4.28% vs. 6.33 ± 3.12%). Morphological observation showed that many embryos appeared blocked at the two- to four-cell stage in KD group (Figure 3B, asterisk).

**TABLE 3:** The developmental potential of early embryos depleted of SIN3A in pigs.

Groups	No. replicates	No. oocytes	No. embryos cleaved (%, mean ± SEM)^*a*^	No. of blastocysts (%, mean ± SEM)^*b*^
NC	5	94	79 (83.63 ± 3.63)	29 (36.64 ± 4.28)^*a*^
KD	5	87	70 (80.08 ± 4.66)	5 (6.33 ± 3.12)^*b*^

**^*a*^The number of embryos cleaved on Day 2 out of the total number of oocytes injected. ^*b*^The number of blastocysts out of the total number of oocytes injected*.*

To further confirm the observation described above and determine which stage the KD embryos arrest, we evaluated the developmental rate at 42 and 80 h, when porcine embryos are supposed to develop to four- and eight-cell stage, respectively. After 42 h, 59.07% of the embryos in the NC group developed to four-cell stage; however, only 14.65% of the embryos in the KD group developed to four-cell stage and most of the them remained at the two-cell stage (Table 4; NC vs. KD 33.57 ± 5.08% vs. 71.07 ± 5.24%). Similarly, most of the embryos in the KD group remained at the two-cell stage, while the control embryos continued to develop normally after 80 h culture (data not shown). These results collectively indicated that depleting SIN3A caused porcine early embryonic developmental block at the two-cell stage.

**TABLE 4:** SIN3A depletion results in two-cell arrest in porcine early embryos.

Groups	No. replicates	No. oocytes	No. 1-cell (%, mean ± SEM)	No. 2-cell (%, mean ± SEM)	No. of 4-cell (%, mean ± SEM)
NC	3	68	5 (7.35 ± 1.47)	23 (33.57 ± 5.08)^*a*^	40 (59.07 ± 3.82)^*a*^
KD	3	73	10 (14.28 ± 6.49)	52 (71.07 ± 5.24)^*b*^	11 (14.65 ± 3.99)^*b*^

**Developmental rates were recorded at 42 h postparthenogenetic activation*. Different letter means statistical significance.*

The phenotypes described above is far more severe than the one we observed for mouse embryos (Zhao et al., 2019), raising the possibility that SIN3A plays a species-specific role during early embryonic development. To further test this possibility, we then examined the functional role of SIN3A using bovine model. Results displayed a successful and efficient KD efficiency of siRNAs after microinjection (Figure 3C). However, there is no significant change in the developmental potential of bovine early embryos (Figure 3D).

### Effect of SIN3A Depletion on Porcine Early Embryonic Development Is Not Solely Dependent on HDAC1/2

**FIGURE 4:**
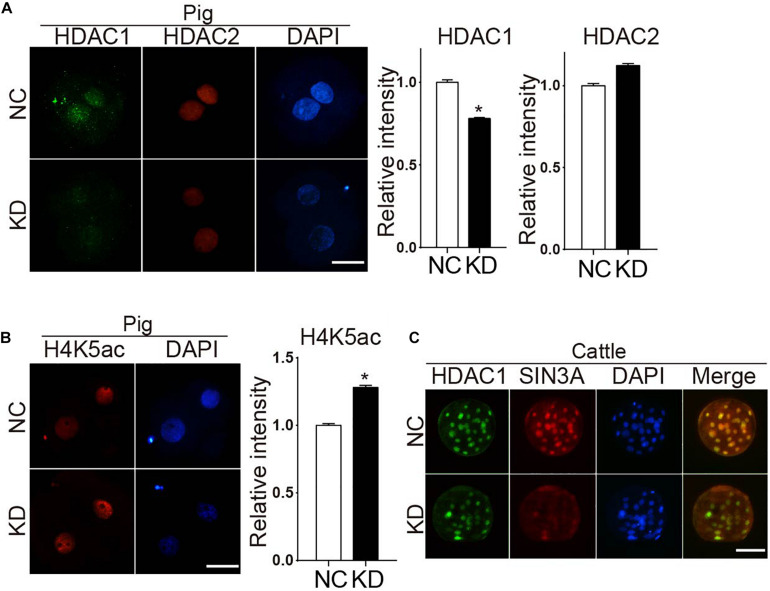
Knockdown of SIN3A results in a slight decrease of HDAC1 in porcine two-cell embryos but not affect the expression of HDAC1 in bovine morula embryos. **(A)** Immunofluorescence staining was performed to analyze the levels of HDAC1 and HDAC2 signal in SIN3A KD and NC groups at two-cell stage. Representative fluorescence images are shown (left). HDAC1 (green), HDAC2 (red), nuclei (blue). Average value of fluorescence intensities of HDAC1 and HDAC2 was assessed by densitometry (right). The experiment was independently repeated three times, and data was indicated as mean ± SEM. *P < 0.05. **(B)** The level of H4K5ac after SIN3A KD was detected by immunofluorescence staining in porcine two-cell embryos (left). H4K5ac (red), nuclei (blue). Quantitative analysis of fluorescence values showed that the expression level of H4K5ac was significantly reduced in pig two-cell embryos with SIN3A knockdown (right). The experiment was independently repeated three times, and data was indicated as mean ± SEM. *P < 0.05. **(C)** Immunofluorescence staining results of the HDAC1 in the bovine morula embryos from NC and SIN3A-KD groups. HDAC1 (green), SIN3A (red), nuclei (blue).

We previously demonstrated that Sin3a regulates mouse early embryonic development through controlling Hdac1 expression. Thus, we examined if HDAC1 were affected in porcine SIN3A-deficient embryos. RNA-seq data revealed no change in both *HDAC1* and *HDAC2* mRNA level in SIN3A-depleted two-cell embryos (Supplementary Figure 2). Immunocytochemical results showed a slight but significant decrease in HDAC1 (*P* < 0.05), and no obvious difference in HDAC2 level (Figure 4A). In addition, H4K5ac level was improved dramatically (Figure 4B).

The slight decrease in HDAC1 observed in pigs could be due to the different stage (two-cell) examined compared with mouse work (morula). Because SIN3A-depleted porcine embryos could not develop to morula stage, we took advantage of the bovine model for which SIN3A-depleted embryos develop to morula stage normally. Results obviously showed that HDAC1 level was not affected whereas endogenous SIN3A protein level was reduced as expected after siRNA treatment in bovine morula embryos (Figure 4C). Collectively, these results suggest SIN3A does not universally regulate HDAC1 expression in early embryos across species.

**FIGURE 5:**
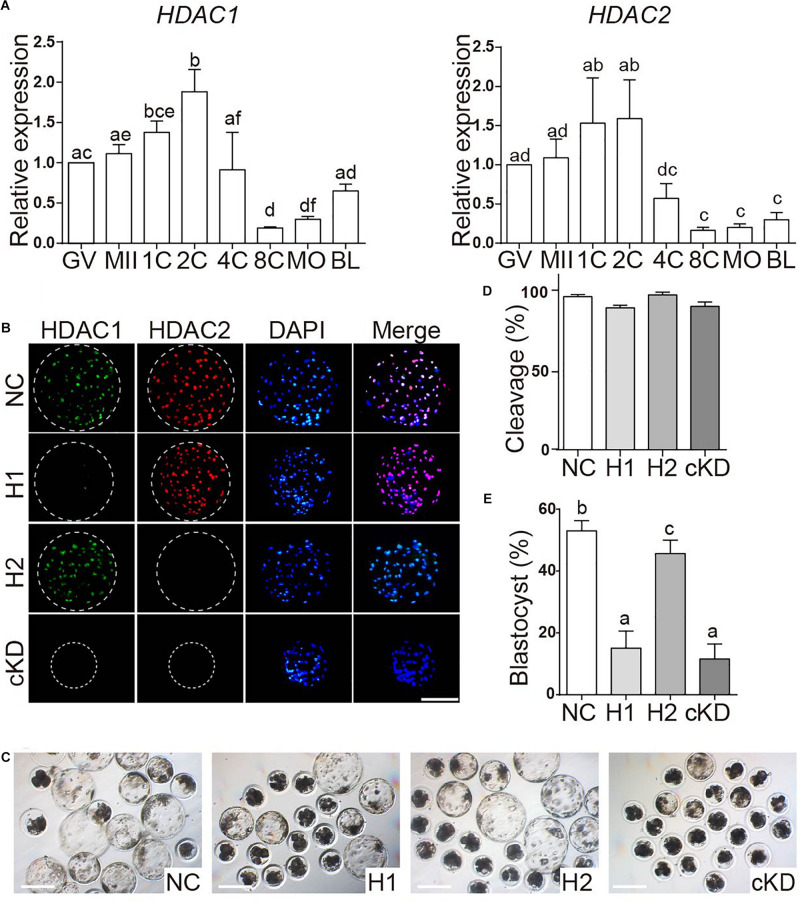
Effect of HDAC1 and HDAC2 KD on the developmental efficiency of porcine embryos. **(A)** Expression of HDAC1 and HDAC2 mRNA in oocytes and early embryos. Relative abundance of HDAC1 and HDAC2 was determined by qPCR. Data are shown as mean ± SEM and different letters on the bars indicated significant differences (*P* < 0.05). **(B)** Validation of the efficiency of RNAi-mediated HDAC1/HDAC2 KD in porcine early embryos. Immunofluorescence analysis of HDAC1 and HDAC2 protein abundance in blastocysts. HDAC1 (green), HDAC2 (red), nuclei (blue). NC, negative control siRNA; H1, *HDAC1* siRNA; H2, *HDAC2* siRNA; cKD, *HDAC1* and *HDAC2* siRNA. **(C)** Representative images of NC, HDAC1 KD, HDAC2 KD, and cKD porcine embryos after *in vitro* culture for 7 days. **(D)**. **(E)** Developmental rates of porcine early embryos. Three independent replicates; all data were represented as mean ± SEM and different letters on the bars indicate significant differences (*P* < 0.05).

SIN3A has been believed to recruit histone deacetylases HDAC1 and HDAC2 to regulate chromatin accessibility and thus gene expression. It prompted us to ask if HDAC1/2 mediated the phenotype of SIN3A depletion on porcine early embryos. First, the expression profiles of *HDAC1* and *HDAC2* mRNA in porcine oocyte and early embryo were characterized. Both HDAC1 and HDAC2 were readily detected throughout oocyte maturation and early embryonic development in pigs. Specifically, mRNA levels in GV and MII oocyte were not different, increased after fertilization and peaked at two-cell stage. Upon EGA, the mRNA abundance gradually decreased, and the level was relatively stable from the eight-cell to the blastocyst stage (Figure 5A).

Next, we tested if the embryos had the same phenotype as *SIN3A* depletion by knocking down HDAC1 and HDAC2, alone or together. Immunofluorescence results confirmed the efficacy of siRNAs targeting HDAC1 or HDAC2 in porcine blastocysts (Figure 5B). Embryo culture results showed cleavage rate was normal, however, blastocyst formation was severely affected in embryos deficient of HDAC1 alone or both HDAC1 and HDAC2 (Figures 5C–E). Collectively, these data suggest that the developmental arrest caused by SIN3A KD was not dependent on HDAC1/2, and other potential mechanisms were involved.

### *CCNB1* Transcript Level Was Significantly Reduced in SIN3A-Deficient Porcine Two-Cell Embryos

**FIGURE 6:**
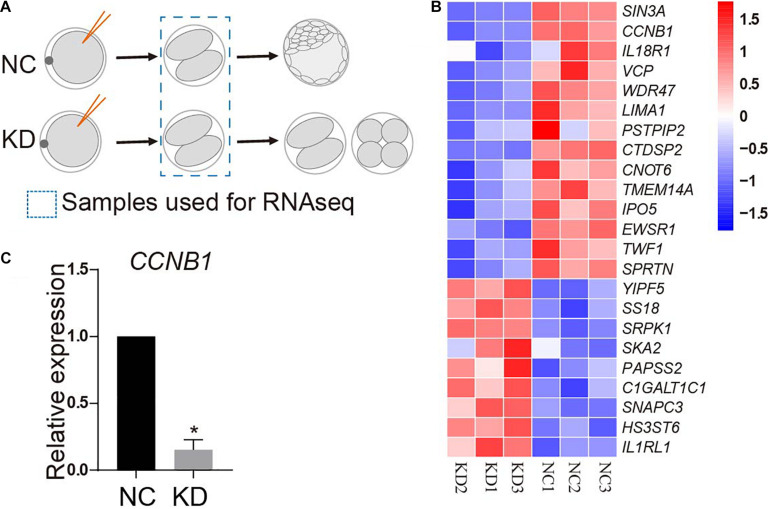
RNA-seq analysis of porcine two-cell embryos deficient of SIN3A. **(A)** Schematic representation of the experimental design. MII oocytes were injected with NC or SIN3A siRNA followed by parthenogenetic activation. Two-cell embryos in each group after being cultured for 32 h were collected for RNA-seq. **(B)** Heatmap showing different expression genes (DEGs) between NC and KD embryos. Three independent replicates were performed. **(C)** Validation of the expression of *CCNB1* mRNA in NC and KD two-cell embryos by qPCR. Three independent replicates; all data were represented as mean ± SEM, *P < 0.05.

To unbiasedly explore the mechanisms underlying the developmental failure associated with SIN3A-depleted embryos, we compared global transcript content between NC and KD two-cell by RNA-seq. Samples were collected at 32 h after parthenogenetic activation prior to the emergence of the morphological difference between two groups (Figure 6A). A significant change in transcript level was observed with 14 gene transcripts increased and nine gene transcripts decreased in KD groups. As expected, RNA-seq analyses proved that SIN3A mRNA abundance was reduced by 80% in KD groups relative to NC. Interestingly, we found that the transcript abundance of *CCNB1*, the gene encoding Cyclin B1, was reduced as well (Figure 6B), which is further confirmed by qPCR assay (Figure 6C).

### CCNB1-Deficient Embryos Mimic the Phenotype of SIN3A-Depleted Embryos in Pigs

**FIGURE 7:**
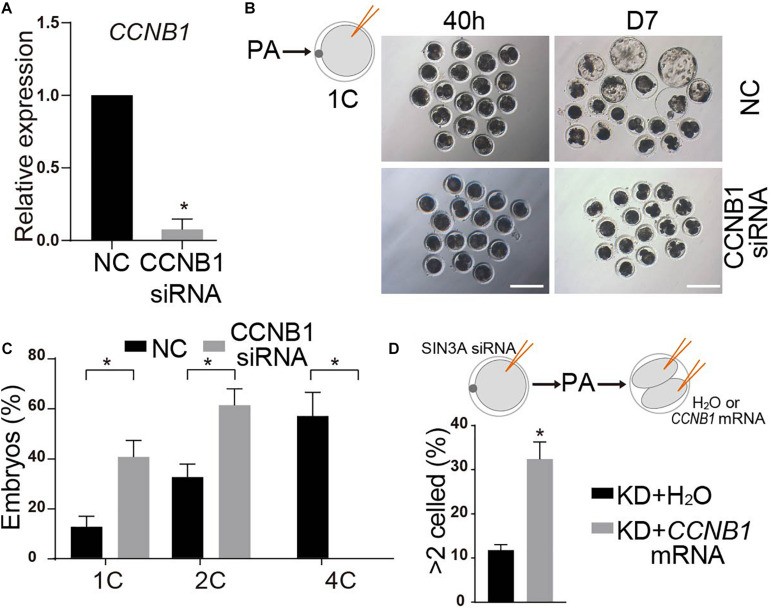
*CCNB1* deficiency leads to two-cell block in SIN3A knockdown embryos. **(A)** qPCR validation of the knockdown efficiency of *CCNB1* siRNA. Embryos were collected at 32 h after parthenogenetic activation. The experiments were independently repeated three times. Values are shown as mean ± SEM, *P < 0.05. **(B)** NC-siRNA or CCNB1-siRNA was introduced into 1C embryos (19 h after parthenogenetic activation) and the representative images of embryos at D7 are shown. **(C)** The developmental rates of two-cell and four-cell embryos were recorded at 40 h after parthenogenetic activation according to the experiment shown in **(B)**. The experiment was independently repeated three times, all data were represented as mean ± SEM, *P < 0.05. **(D)** H_2_O or *CCNB1* mRNA were microinjected into both blastomere of SIN3A-depleted two-cell embryos, and then the percentage of embryos developing beyond two cells was counted. The experiment was independently repeated three times, all data were represented as mean ± SEM, *P < 0.05.

CCNB1 has been established as a critical regulator of cell cycle progression during oocyte maturation and early embryonic development in mammals (Zhang et al., 2010; Strauss et al., 2018). Therefore, we speculated that SIN3A regulated early embryonic development *via* modulation of *CCNB1* expression in pigs. To test this hypothesis, we first asked if CCNB1 KD could mimic the phenotype of SIN3A depletion. Surprisingly, all the embryos failed to cleave when we injected *CCNB1* siRNAs into MII oocytes. This developmental failure could be attributed to the functional requirement of CCNB1 on the meiotic or mitotic progression. Then, we chose to inject *CCNB1* siRNA at the late phase of pronuclear stage (19 h after parthenogenetic activation). QPCR results showed that siRNAs can effectively reduce the level of endogenous *CCNB1* mRNA as expected (Figure 7A). Embryo culture data revealed that nearly all the embryos deficient of CCNB1 were arrested at two-cell stage and few develop to the four-cell stage (Figures 7B,C), reminiscent of the phenotype of SIN3A-depleted embryos.

Then we wondered whether the addition of exogenous *CCNB1* mRNA into SIN3A KD embryos could restore the early embryonic development. To avoid the potential negative posttranscriptional effects of SIN3A KD on *CCNB1* mRNA, we injected mouse *CCNB1* mRNA, which is highly homologous with porcine (Zhang et al., 2010). Because CCNB1 is subject to periodical posttranslational regulation through each cell cycle (Bouftas and Wassmann, 2019), we injected *SIN3A* siRNA into the MII stage oocytes and then injected the CCNB1 mRNA into the two blastomeres. Results showed that the injection of *CCNB1* mRNA into both blastomeres significantly improve the percentage of embryos developing beyond two-cell stage relative to control groups (Figure 7D). Altogether, these data suggest that SIN3A regulates porcine early embryonic development partly through regulation of CCNB1 expression.

## Discussion

SIN3/HDAC chromatin repressor complex is involved in the regulation of a variety of biological processes, including cell proliferation and differentiation, apoptosis, and cell cycle progression (Halder et al., 2017; Saunders et al., 2017; Yao et al., 2017; Ren et al., 2019). Recently, we have demonstrated critical roles of key components of SIN/HDAC complex, including SUDS3 (Zhang et al., 2013), HDAC1/2 (Zhao et al., 2020), and SIN3A (Zhao et al., 2019), in mouse early embryonic development. However, the related mechanisms and its functional conservation among species have been largely unresolved. To dissect the mechanism of SIN3A function in early embryos, we found SIN3A regulates the development progression through the morula-to-blastocyst transition *via* HDAC1 in mouse embryos (Zhao et al., 2019). However, herein, we found alternative functional roles of SIN3A as well as potential mechanisms using porcine model.

In the present study, it was found that the porcine early embryos with SIN3A deletion were blocked at two-cell stage, which is more severe than the developmental defects found in mouse, suggesting species-specific role of SIN3A in early embryonic development. Furthermore, in contrast with our previous mouse data, SIN3A depletion did not trigger dramatical reduction in HDAC1 mRNA and protein level in both porcine and bovine early embryos, further consolidating the species-specific effects of SIN3A. In addition, knocking down HDAC1 and HDAC2 alone or together did not phenocopy SIN3A ablation in porcine embryos. We also identified that *CCNB1* mRNA level was significantly reduced upon SIN3A depletion. CCNB1 KD lead to two-cell block, similar with the phenotype of SIN3A depletion. Microinjection of the exogenous *CCNB1* mRNA into the SIN3A-deleted two-cell embryo could partly restore development beyond two-cell stage. Thus, we believe that SIN3A is required for porcine early embryonic development through maintaining CCNB1 expression.

Maternal factors stored during oocyte growth and maturation, including mRNAs and proteins, are crucial for biological events such as EGA (De Iaco et al., 2017; Hendrickson et al., 2017), chromatin reprogramming (Inoue et al., 2017; Kannampuzha-Francis et al., 2017), and maintenance of genome stability (Xu et al., 2017). We previously demonstrated the functional importance of key maternal factors during porcine early embryonic development. For example, WDR5 is required to foster porcine early embryonic development probably *via* regulating key epigenetic modifications and genome integrity (Ding et al., 2017). Here, we found SIN3A mRNA abundance was dramatically lower in post-EGA embryos than pre-EGA embryos among all species examined, suggesting a conserved maternal origin of SIN3A. However, it was noticed the protein level did not changed correspondingly as the mRNA level of SIN3A, suggesting a posttranscriptional regulation of *SIN3A* mRNA or posttranslational regulation of SIN3A protein in early embryos. Indeed, it has been shown that SIN3A is a dormant maternal mRNA recruited during oocyte maturation and early development and SIN3A protein is subject to the proteasome-dependent degradation in mouse early embryos (Jimenez et al., 2015).

SIN3A protein abundance peaks at four-cell stage during porcine early embryonic development, suggesting a critical role of SIN3A in the development through four-cell stage. Our functional evidence indicates SIN3A depletion in pig embryos resulted in a developmental arrest at two-cell stage (prior to EGA) in pigs. To our knowledge, this is one of the most severe defects when ablating a specific gene function in porcine early embryos. As genome-wide transcriptional activity is generally believed silent in porcine two-cell embryos, it is plausible that the maternal control of early development is disrupted in SIN3A-deficient embryos, thus leading to developmental failure prior to EGA. Similarly, depletion of maternal SIN3A *via* RNAi in oocytes causes the failure to develop beyond two-cell stage, the mechanism of which remains unclear (Jimenez et al., 2015). However, depleting SIN3A by injecting siRNAs into zygotes permit embryos developing to morula stage but not blastocyst stage in mice (Zhao et al., 2019). This discrepancy could be attributed to that injection at zygote stage could not leave enough time for siRNAs to deplete maternal mRNA. Thus, these studies suggest SIN3A is a critical maternal-effect gene in pigs.

SIN3/HDAC complex has conventionally been thought as a genome-wide transcriptional repressor *via* recruiting HDAC1/2 (Tiana et al., 2018; Gambi et al., 2019; Monteleone and Poli, 2019). Inhibiting maternal Sin3a mRNA during oocyte maturation reduces the level of H3K18ac, H4K8ac, and H4K12ac, except H4K5ac level in mouse one-cell embryos (Jimenez et al., 2015). Meanwhile, Sin3a depletion results in a dramatical decrease in Hdac1 mRNA and protein level in mouse morula. However, in the present work, we observed a slight decrease in HDAC1 level in SIN3A-deficient porcine two-cell embryos. We originally attributed this difference to the different embryo stages used in two studies (morula and two-cell). However, our further study in cattle revealed that SIN3A depletion caused no change in HDAC1 at morula stage, consolidating our conclusion that SIN3A’s role in mammalian early development is species dependent. An alternative possible explanation for this observation is that EGA occurs late in porcine and bovine early embryos compared with mouse and the abundant maternal HDAC1 mRNA may prohibit us from observing the effect of SIN3A depletion on HDAC1 expression.

We further reasoned that SIN3A may function through regulating HDAC1 activity but not affecting its protein level. Indeed, we found there is a significant increase in H3K5ac upon SIN3A depletion in pigs. However, if we ablated HDAC1 and/or HDAC2 individually or together, embryos could develop beyond two-cell stage although HDAC1 KD or HDAC1/2 co-KD decreased the potential of embryos developing to blastocyst stage. These results rule out the possibility that SIN3A work through HDAC1/2 to control development through two-cell stage in pigs. In addition, the requirement of HDAC1 for blastocyst formation in pigs documented here is in contrast with the observation that HDAC1 alone is dispensable for mouse preimplantation development as we described before (Zhao et al., 2020). However, HDAC1 and HDAC2 are functional redundant during mouse preimplantation development (Zhao et al., 2020). Thus, it warrants further investigation on the mechanism of the differential HDAC1 effects in mammalian early development.

The precise regulation of cell cycle depends on the complex containing Cyclin and Cyclin-dependent kinase (Palmer and Kaldis, 2016). For example, the CDK1/Cyclin B complex is involved in the regulation G2/M conversion process (Kellogg, 2003). Cyclin B and its protein kinase CDK1 are also components of the maturation promoting factor (MPF) complex, which regulates oocytes to resume meiosis and promote oocyte maturation (Holt et al., 2013). The proper expression of Cyclin B and other cell cycle factors is essential for oocyte maturation and early embryo development. The embryos of Cyclin B1^–/–^ mice are arrested in the second mitotic G2 phase (Strauss et al., 2018); Cyclin B2^–/–^ mice exhibit premature ovarian failure and oocytes are unable to mature (Daldello et al., 2019). The developmental competence of porcine early embryos is also positively associated with *CCNB1* level (Zhang et al., 2010). Here, we found CCNB1 mRNA abundance is significantly reduced upon SIN3A depletion in porcine early embryos. Thus, we speculated that the developmental arrest at two-cell stage observed in SIN3A-deficient porcine embryos is attributed to the aberrant *CCNB1* expression. The importance of the maternal control of cell cycle is also observed while chromatin remodeling factor Snf2h is deleted in mouse oocytes with meiosis progression inhibited, likely due to failure of MPF activation (Zhang et al., 2020). Interestingly, the exogenous *CCNB1* mRNA can restore the meiosis (Zhang et al., 2020). Therefore, the cell cycle progression is tightly under control by maternal stored factors, including SIN3A documented here. However, it requires further investigation about the mechanism underlying the regulation of SIN3A on *CCNB1* expression.

CCNB1 mRNA is present at low level in porcine GV oocytes, but its abundance increases dramatically after oocyte maturation, maintains throughout four-cell stage and drops sharply to low level thereafter, exhibiting a typical expression pattern of maternal-effect genes (Zhang et al., 2010). Cytoplasmic polyadenylation is a well-known mechanism involved in protecting maternal mRNAs from degradation (Sha et al., 2019). It has been shown there is a significant change on the polyadenylation length at the 3′-untranslated region (3′-UTR) of *CCNB1* mRNA during porcine early embryonic embryos, suggesting CCNB1 is regulated posttranscriptionally *via* cytoplasmic polyadenylation during oocyte maturation (Zhang et al., 2010). We speculated that SIN3A is involved, maybe indirectly, in the protection of the polyadenylation length of *CCNB1* mRNA. Therefore, whether maternal SIN3A protects it from degradation through maintaining the length of *CCNB1* mRNA 3′-UTR warrants exploration.

## Conclusion

The present work demonstrated that the expression of SIN3A exhibits the pattern of a typical maternal-effect gene during mammalian early embryonic development. Our functional evidence in mouse, porcine, and bovine embryos collectively demonstrates the species-specific role of SIN3A in early embryonic development. Importantly, we determined that SIN3A depletion triggered a significant reduction in *CCNB1* mRNA level in pigs. Functional work further proved that CCNB1 KD embryos phenocopied SIN3A-depleted embryos whereas the exogenous *CCBN1* mRNA could partly rescue the SIN3A-deficient embryos to pass through two-cell stage. Thus, we believe that SIN3A is required for porcine early embryonic development, likely through the regulation of *CCNB1* expression.

## Data Availability Statement

The datasets generated for this study can be found in online repositories. The names of the repository/repositories and accession number(s) can be found below: https://www.ncbi.nlm.nih.gov/geo/, GSE157650.

## Ethics Statement

The animal study was reviewed and approved by Laboratory Animal Center, Zhejiang University.

## Author Contributions

KZ and LL conceived and designed the experiments. LL performed porcine embryo production and microinjection in porcine oocytes. LL and YS performed bovine IVP, microinjection, and embryo staining. YD participated in the RNAseq library construction and bioinformatic analysis. LL, YD, PZ, and KZ participated in data analysis. LL and KZ wrote the manuscript. YZ was responsible for coordinating the research in pigs. All authors discussed and interpreted the data and approved the manuscript.

## Conflict of Interest

The authors declare that the research was conducted in the absence of any commercial or financial relationships that could be construed as a potential conflict of interest.

## Footnotes

1 https://combine-lab.github.io/salmon/

2 https://ccb.jhu.edu/software/hisat2

3 http://cole-trapnell-lab.github.io/cufflinks/

## Supplementary Material

Supplementary Figure 1 Validation of the specificity of SIN3A antibody. Mouse zygotes were microinjected of either *Cas9* mRNA (control) or *Sin3a* mRNA, and two-cell embryos were collected and subject to immunofluorescence analysis. Scale bar = 50 μM.

Supplementary Figure 2 HDAC1 and HDAC2 mRNA level was not changed after SIN3A knockdown as determined by RNA-seq.
