# Supplementary figures and images for: SIN3A Regulates Porcine Early Embryonic Development by Modulating CCNB1 Expression
Source: Front Cell Dev Biol. 2021 Feb 22;9:604232. doi: 10.3389/fcell.2021.604232 (PMC7937639; doi:10.3389/fcell.2021.604232)

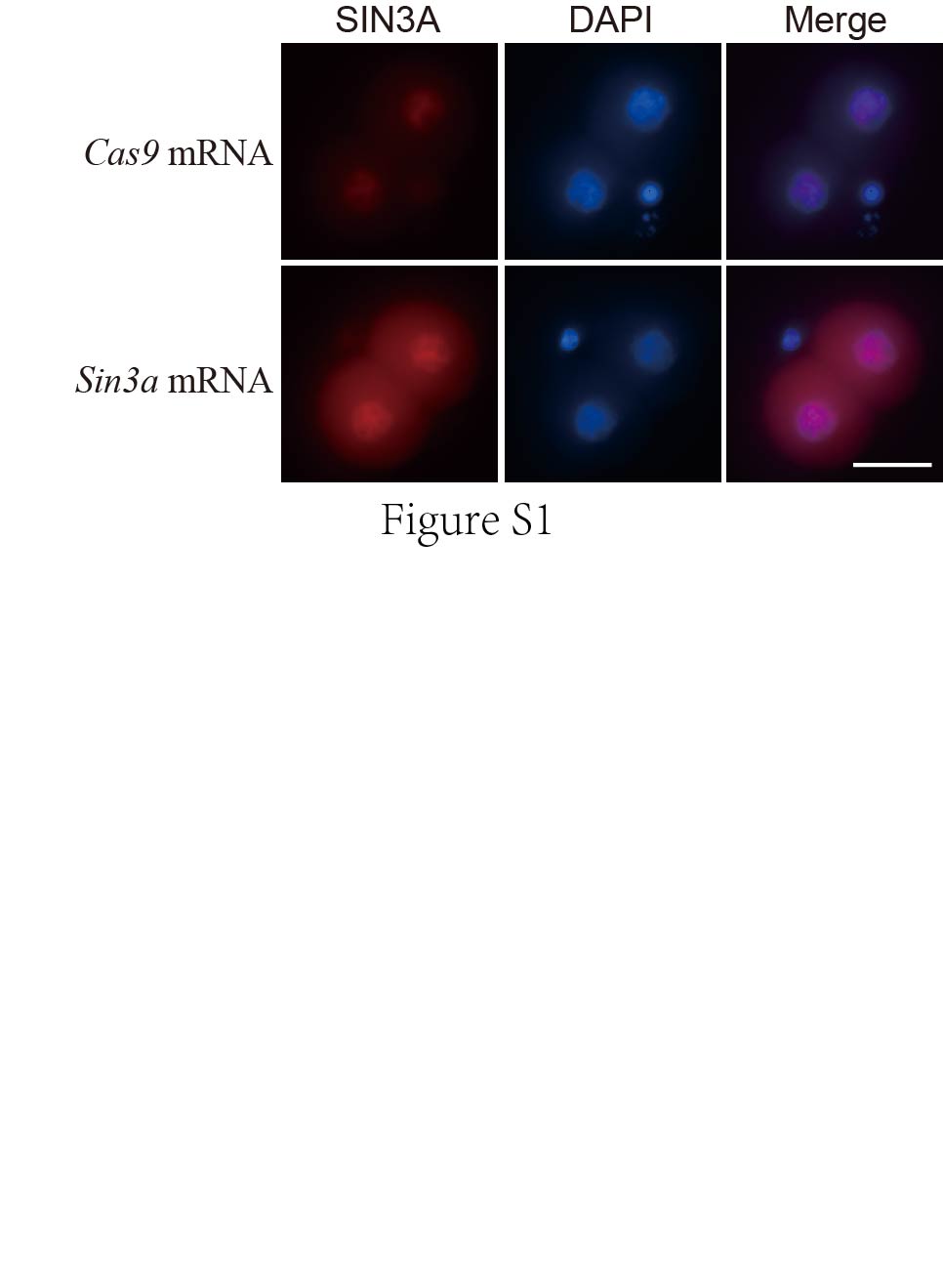

Supplement: Supplementary Figure 1 — Validation of the specificity of SIN3A antibody. Mouse zygotes were microinjected of either Cas9 mRNA (control) or Sin3a mRNA, and two-cell embryos were collected and subject to immunofluorescence analysis. Scale bar = 50 μM. [file Image_1.jpg]

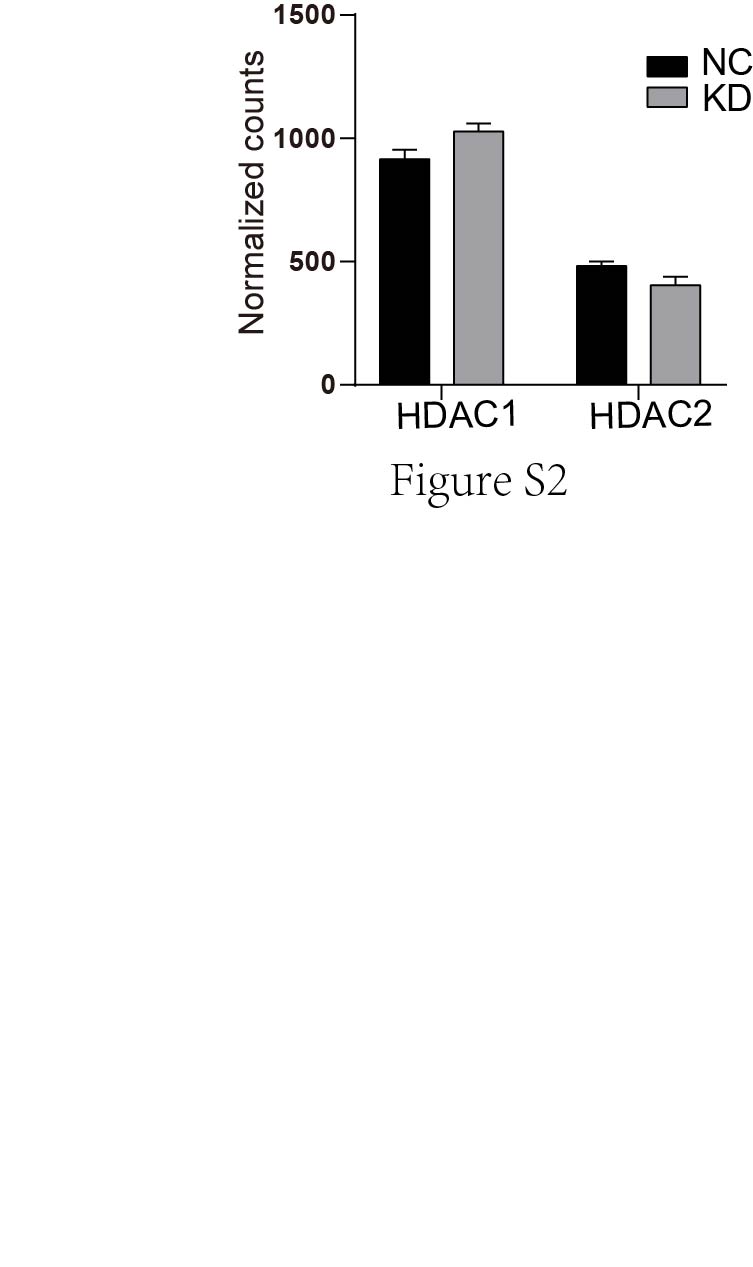

Supplement: Supplementary Figure 2 — HDAC1 and HDAC2 mRNA level was not changed after SIN3A knockdown as determined by RNA-seq. [file Image_2.jpg]
